# The femoral anterior tangent line could serve as a reliable alternative reference axis for distal femoral rotational alignment in total knee arthroplasty: an MRI-based study

**DOI:** 10.3389/fsurg.2024.1363551

**Published:** 2024-03-14

**Authors:** Shuzhen Li, Haiquan Deng, Lianjian Jiang, Haibo Liang, Jianchao Sun, Youjia Xu

**Affiliations:** ^1^ Department of Orthopaedic Surgery, The Second Affiliated Hospital, Soochow University, Suzhou, China; ^2^ Department of Orthopaedic Surgery, Guangxi Hospital Division of the First Affiliated Hospital, Sun Yat-sen University, Nanning, China; ^3^ Department of Joint and Sports Medicine Surgery, The People’s Hospital of Guangxi Zhuang Autonomous Region, Nanning, China; ^4^ Graduate School, Youjiang Medical University for Nationalities, Baise, China

**Keywords:** total knee arthroplasty, femoral anterior tangent line, rotational alignment, magnetic resonance imaging, lower limb alignment

## Abstract

**Background:**

This study aimed to evaluate the reference value of the femoral anterior tangent (FAT) line as a guidance of distal femoral rotation on magnetic resonance images (MRI).

**Methods:**

We retrospectively included 81 patients (106 knees) diagnosed as ailing from primary knee osteoarthritis. The indirect rotational axes including the FAT line, the perpendicular line to the anteroposterior axis (pAPA), and the posterior condylar axis (PCA) were identified on MRI, and their angles related to the clinical transepicondylar axis (cTEA) or surgical transepicondylar axis (sTEA) were measured. The patients were further divided into subgroups according to the Kellgren–Lawrence (K–L) grades, the joint-line convergence angle (JLCA), and the arithmetic hip-knee-ankle angle (aHKA) to assess the variance of different rotational reference axes.

**Results:**

The FAT line was −11.8° ± 3.6° internally rotated to the cTEA and −7.5° ± 3.6° internally rotated to the sTEA. The FAT/cTEA angle and the FAT/sTEA angle shared a similar frequency distribution pattern but a little greater variance to the pAPA/cTEA angle and the PCA/cTEA angle. The PCA/cTEA angle in the JLCA |*x*| ≥ 6° subgroup was significantly smaller than in the two other JLCA subgroups. The pAPA/cTEA angle and the PCA/cTEA angle also presented statistical significance within the aHKA subgroups. While the FAT/cTEA angle and the FAT/sTEA angle demonstrated superior stability among the different K–L grades, JLCA subgroups, and aHKA subgroups.

**Conclusion:**

The FAT line was less affected by the degree of knee osteoarthritis and lower limb alignment, which could serve as a reliable alternative reference axis for the distal femoral rotational alignment in total knee arthroplasty.

## 1 Introduction

The rotational alignment of the femoral component plays a critical role in total knee arthroplasty (TKA) (1, 2). Proper rotational alignment of the femoral component can reduce wear of the prosthesis, decrease patellofemoral joint instability and pain, and extend the lifetime of the prosthesis (3–5). To obtain a good rotational alignment of the femoral prosthesis, there are currently several femoral axes that can be used as references during surgery, including the surgical transepicondylar axis (sTEA), the clinical transepicondylar axis (cTEA), the posterior condylar axis (PCA), and the perpendicular line to the anteroposterior axis (pAPA) (6–8). The sTEA is considered to be closer to the rotational axis of the distal femur, but the sulcus of the medial epicondyle cannot be detected in all patients (9). In addition, due to the obstruction of the soft tissues, it is often difficult to expose the medial and lateral epicondylar prominence during TKA, which limits the measurement of cTEA (10, 11). To solve this problem, the PCA and the pAPA are recommended as indirect references for distal femoral rotation (12). The PCA is generally internally rotated 3° with respect to the sTEA, and the pAPA is considered perpendicular to the sTEA (13). However, in patients with osteoarthritis, cartilage wear and subchondral bone deformation are common. Posterior condyle cartilage wear can lead to inaccurate PCA measurement and trochlear dysplasia, or cartilage wear may distract the pAPA from the orthodox position, both of these situations could lower the reference value of the PCA and the pAPA (14, 15).

Talbot and Bartlett used a computer navigation system to measure the anterior surface of the femur during surgery, and proposed that the femoral anterior tangent (FAT) line could serve as a new reference axis for the rotational alignment of the distal femur (16). The FAT line is a tangent line to the anterior surface of the femur immediately proximal to the trochlea. Watanabe et al. measured the angle between the FAT line and the cTEA on computed tomography (CT) and compared it with the angles of the pAPA and the PCA relative to the cTEA. They found that the FAT line possessed a comparable stability to the pAPA, better than the PCA (17). However, Watanabe et al. analyzed the reference axes on CT images, which might ignore the influence of cartilage wear of the femoral condyle (18), and all of their research subjects were patients underwent TKA, whether the FAT line is applicable in patients with mild knee osteoarthritis remains unknown.

Based on the aforementioned issues, this study intended to evaluate the reference value of the FAT line as a guidance of distal femoral rotation on magnetic resonance imaging (MRI). We further grouped our research subjects according to the degree of knee osteoarthritis and the lower limb alignment to assess the variance of different rotational reference axes. We hypothesized that the FAT line was stable on MRI and could be used as a reliable femoral rotational alignment reference axis in TKA.

## 2 Methods

We retrospectively selected continuous patients who came to our hospital for knee joint pain from January 2019 to December 2021. All of the patients were diagnosed as having primary knee osteoarthritis. The patients with fractures around the knee joint, cruciate ligament tear, and infectious diseases were excluded. The selected patients had completed a 3.0 T magnetic resonance scan (Philips, Andover, USA) of the knee joint and a full-length radiograph of lower limbs in standing position (Philips, Andover, USA). This study was approved by institutional review board of the authors’ hospital.

**Figure 1:**
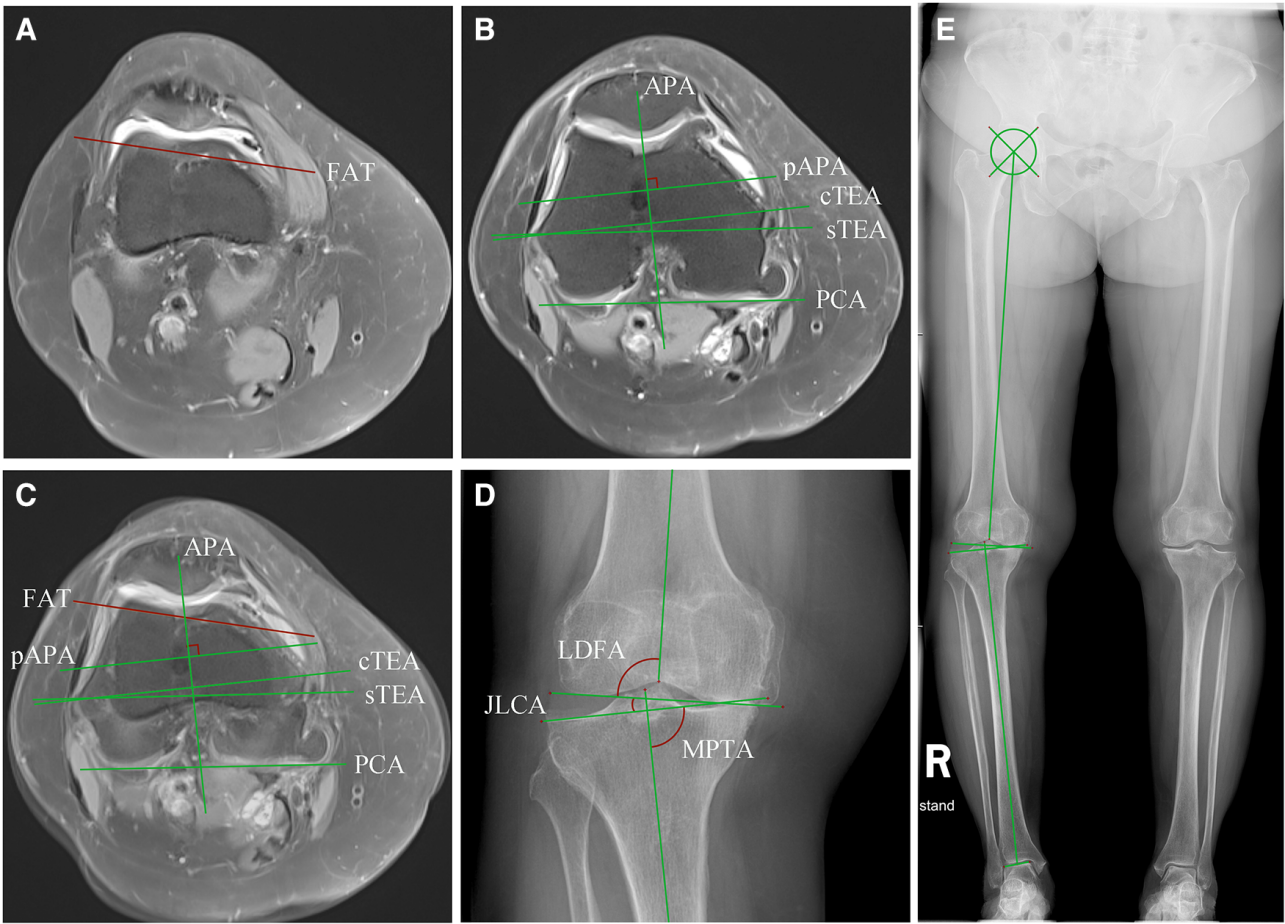
Measurement of the distal femoral rotational axes on MRI and lower limb alignment angles on radiography. (**A**) The FAT line was drawn on the transverse plane of MRI. The slice was usually two slices apart from the upper edge of the trochlear cartilage; (**B**) The cTEA, the sTEA, the PCA, the APA, and the line pAPA were identified on another slice of the transverse MRI. Note that the cartilage wear of the posterior femoral condyle and the trochlear could sometimes affect the position of the PCA and pAPA. (**C**) (**A**) and (**B**) were merged and the angles between the indirect rotational reference axes and the transepicondylar axes were measured. (**D**) The JLCA, the LDFA, the MPTA, and the mHKA were measured on a knee radiograph derived from a double-limb stance radiograph (**E**). The position of the lower limb alignment axes and angles are shown in (**E**).

The images of the MR and radiography were imported into RadiAnt DICOM Viewer (version 2021, Poznan, Poland) in DICOM format, the reference axes were drawn in the software, and the corresponding angles were measured (Figure 1). The FAT line was a tangent line to the anterior femoral cortex immediately above the superior border of the trochlea. The anteroposterior axis (APA, or Whiteside's line) was a line connecting the lowest point of the trochlear groove and the apex of the posterior intercondylar notch, and the pAPA is the perpendicular line to the APA. The PCA was a line connecting the most posterior points of the medial and lateral femoral condyles. The sTEA connected the most prominent point of the lateral femoral epicondyle and the sulcus of the medial femoral epicondyle, and the cTEA connected the most prominent points of the medial and lateral femoral epicondyles. On the double-limb standing radiograph, the line connecting the center of the femoral head to the apex of the intercondylar notch of the femur was the mechanical axis of the femur. The line connecting the midpoint of the intercondylar ridge of the tibia to the midpoint of the upper edge of the talus was the mechanical axis of the tibia. The angle between the mechanical axis of the femur and the mechanical axis of the tibia was the mechanical hip-knee-ankle angle (mHKA). The lateral distal femoral angle (LDFA) was the lateral angle formed by the mechanical axis of the femur and the tangent line of the medial and lateral femoral condyles. The medial proximal tibial angle (MPTA) was defined as the medial angle formed by the mechanical axis of the tibia and the tangent line of the tibial plateau. The joint-line convergence angle (JLCA) was the angle between the knee joint lines of the distal femur and the proximal tibia. The arithmetic hip-knee-ankle angle (aHKA) was the angle calculated by subtracting the LDFA from the MPTA, which reflected the lower limb alignment before the occurrence of knee osteoarthritis (19). The mHKA and aHKA were expressed as negative values for varus genu, and positive values for valgus genu. If the JLCA opening faced outward, the value was positive, and if the opening faced inward, the value was negative. The angles formed by the FAT line, the pAPA, and the PCA in terms of the cTEA were expressed as FAT/cTEA, pAPA/cTEA, and PCA/cTEA, respectively. The angle between the FAT line and the sTEA was expressed as FAT/sTEA. Internal rotation of the aforementioned indirect reference axes relative to the cTEA or the sTEA was designated the negative value, and external rotation was designated the positive value. The severity of knee osteoarthritis was evaluated on radiographs using the Kellgren–Lawrence grading system (K–L grades) in accord with previously published literature (20).

Intra- and inter-observer reliabilities were determined with intraclass correlation coefficients (ICCs) for each measurement. We randomly selected 35 patients and two of the authors (HD and LJ) assessed the relevant axes with a four-week interval. The ICCs of intra- and inter-observer reliability of the preliminary analysis were >0.9. To save time, we assigned one of the authors (HD) to conduct all of the measurements.

The graphs were generated by the GraphPad Prism (version 7.0, CA, USA). Statistical analyses were performed using the SPSS statistic software (version 22.0, New York, USA). The angles between the indirect reference axes and the transepicondylar axes were presented as mean and standard deviation (SD). A two- sample *t*-test was used to compare lower extremity alignment and femoral rotational angles between men and women. The correlations between femoral rotational angles and mHKA were analyzed using the Pearson correlation test. Differences between the subgroups according to the K–L grades, the JLCA, and the aHKA were compared using the analysis of variance (ANOVA) test with the least significant difference test. *P*-value < 0.05 was considered statistically significant.

## 3 Results

A total of 91 patients met our diagnostic criteria and had undergone complete MRI and radiography. Among them, four patients were excluded because their MRIs were blurred and some of the rotational axes could not be identified, another four patients, whose medial sulcus of femoral epicondyle were missing on the MRI, were excluded as well. Two patients were excluded owing to femoral head necrosis, which hampered the measurements of lower limb alignment. Finally, 81 patients (106 knees) were included in our study, the demographic characteristics of the study population are provided in Table 1.

**Table 1:** Demographic characteristics of the study population.

Variables	
Included patients	81
Total knees	106
Age, mean ± SD, (range)	62.2 ± 9.7, (41–82)
Gender, male (%)/female (%)	27 (25.5%)/79 (74.5%)
Measurement side, right/left (%)	60 (56.6%)/46 (43.4%)
Treatment type, *n* (%)	
Conservative^a^	20 (18.9%)
Arthroscopic debridement	34 (32.1%)
HTO	2 (1.9%)
UKA	7 (6.6%)
TKA	43 (40.6%)

HTO, high tibial osteotomy; UKA, unicompartmental knee arthroplasty; TKA, total knee arthroplasty; SD, standard deviation.

^a^ Conservative treatments included exercise, oral or topical application of non-steroidal anti-inflammatory drugs, intra-articular hyaluronic acid injection, and intra-articular platelet-rich plasma injections.

The mechanical axes and knee joint angles of the study population are presented in Table 2. There was no significant difference noted between the men and women patients in terms of mHKA, aHKA, JLCA, and LDFA. However, the MPTA in men was significantly smaller than that in the women (*P* < 0.05). The FAT line was −11.8° ± 3.6° internally rotated to the cTEA and −7.5° ± 3.6° internally rotated to the sTEA. The men demonstrated larger FAT/cTEA and FAT/sTEA angles than the women, while only the FAT/sTEA angle was found to be statistically significant (*P* < 0.05). The average value of the pAPA/cTEA angle was 1.7° ± 2.9°, there was no statistical difference between men and women. The PCA was −6.0° ± 2.2° internally rotated to the cTEA, and the angle was significantly smaller in men than in the women (*P* < 0.05).

**Table 2:** Lower limb alignment angles and distal femoral rotation angles of the study population (*n* = 106)^a^.

Criteria	Whole patients (*n* = 106)	Male (*n* = 27)	Female (*n* = 79)	*P*-value
Lower limb alignment angles				
mHKA^b^	−4.4 ± 6.4	−4.2 ± 5.9	−4.5 ± 6.6	0.840
aHKA^b^	−1.6 ± 4.2	−2.0 ± 4.3	−1.5 ± 4.2	0.622
JLCA^c^	3.0 ± 3.4	2.6 ± 2.7	3.1 ± 3.6	0.501
LDFA	88.7 ± 2.9	88.0 ± 2.4	88.9 ± 3.0	0.159
MPTA	87.1 ± 2.6	**86.0 ± 2.8**	**87.4 ± 2.5**	**0**.018*
Distal femoral rotation angles				
FAT/cTEA^d^	−11.8 ± 3.6	−12.9 ± 3.1	−11.4 ± 3.6	0.067
FAT/sTEA^d^	−7.5 ± 3.6	**−9.0 ± 3.3**	**−7.0 ± 3.5**	**0**.010*
pAPA/cTEA^d^	1.7 ± 2.9	2.5 ± 3.2	1.4 ± 2.7	0.096
PCA/cTEA^d^	−6.0 ± 2.2	**−5.2 ± 1.6**	**−6.3 ± 2.3**	**0**.024*

^a^ Data are presented as mean ± standard deviation (in degrees).

^b^ Negative value signifies varus genus, and positive value signifies valgus genus.

^c^ Negative value signifies the JLCA opening faces inward, and positive value signifies the JLCA opening faces outward.

^d^ Negative value signifies internal rotation relative to the corresponding transepicondylar axis and positive value indicates external rotation.

* Statistical significance between male and female patients.

**Figure 2:**
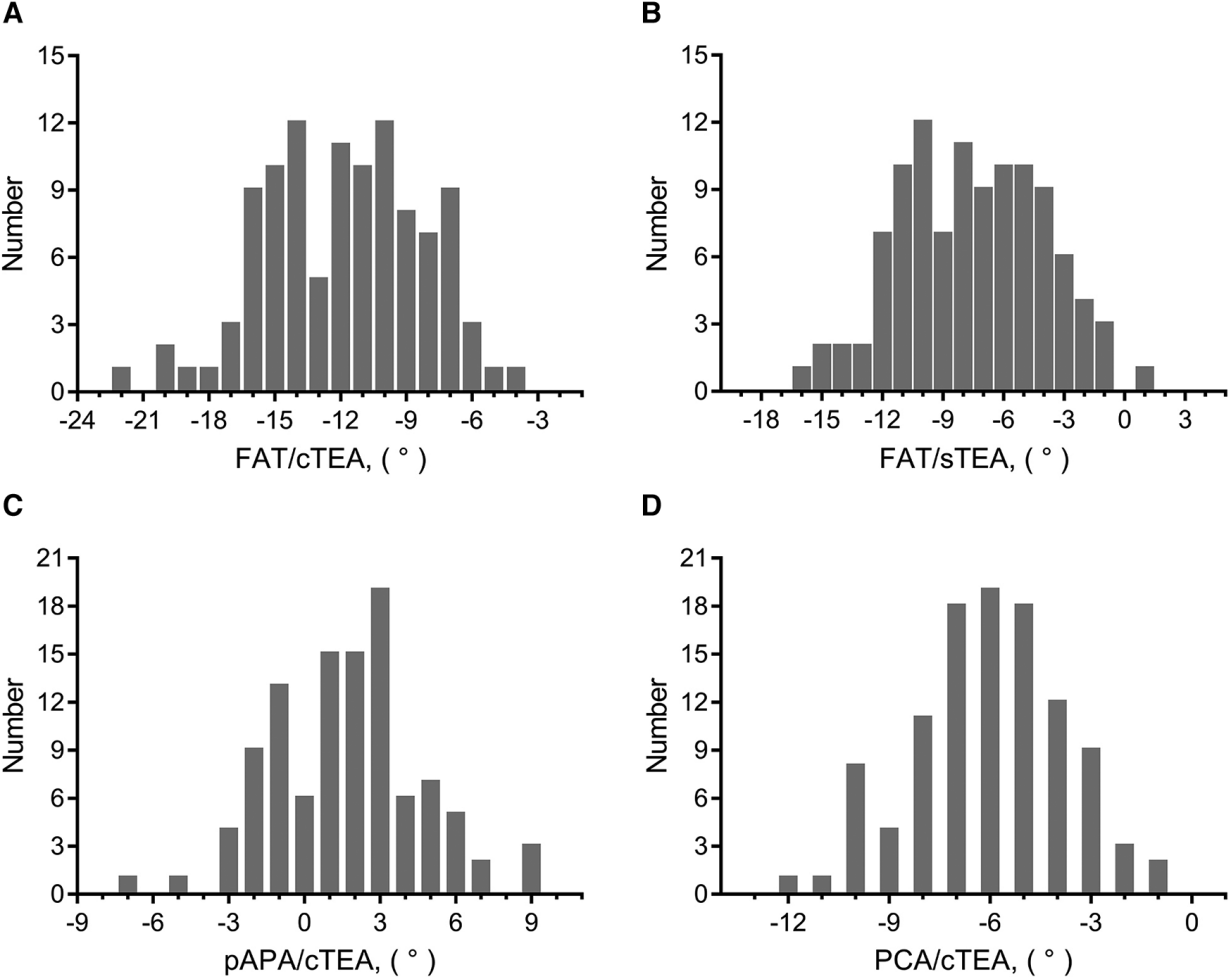
Frequency distribution of femoral rotational angles between the indirect rotational reference axes and transepicondylar axes. (**A**) Frequency distribution pattern of the FAT/cTEA angle. (**B**) Frequency distribution pattern of the FAT/sTEA angle. (**C**) Frequency distribution pattern of the pAPA/cTEA angle. (**D**) Frequency distribution pattern of the PCA/cTEA angle.

**Figure 3:**
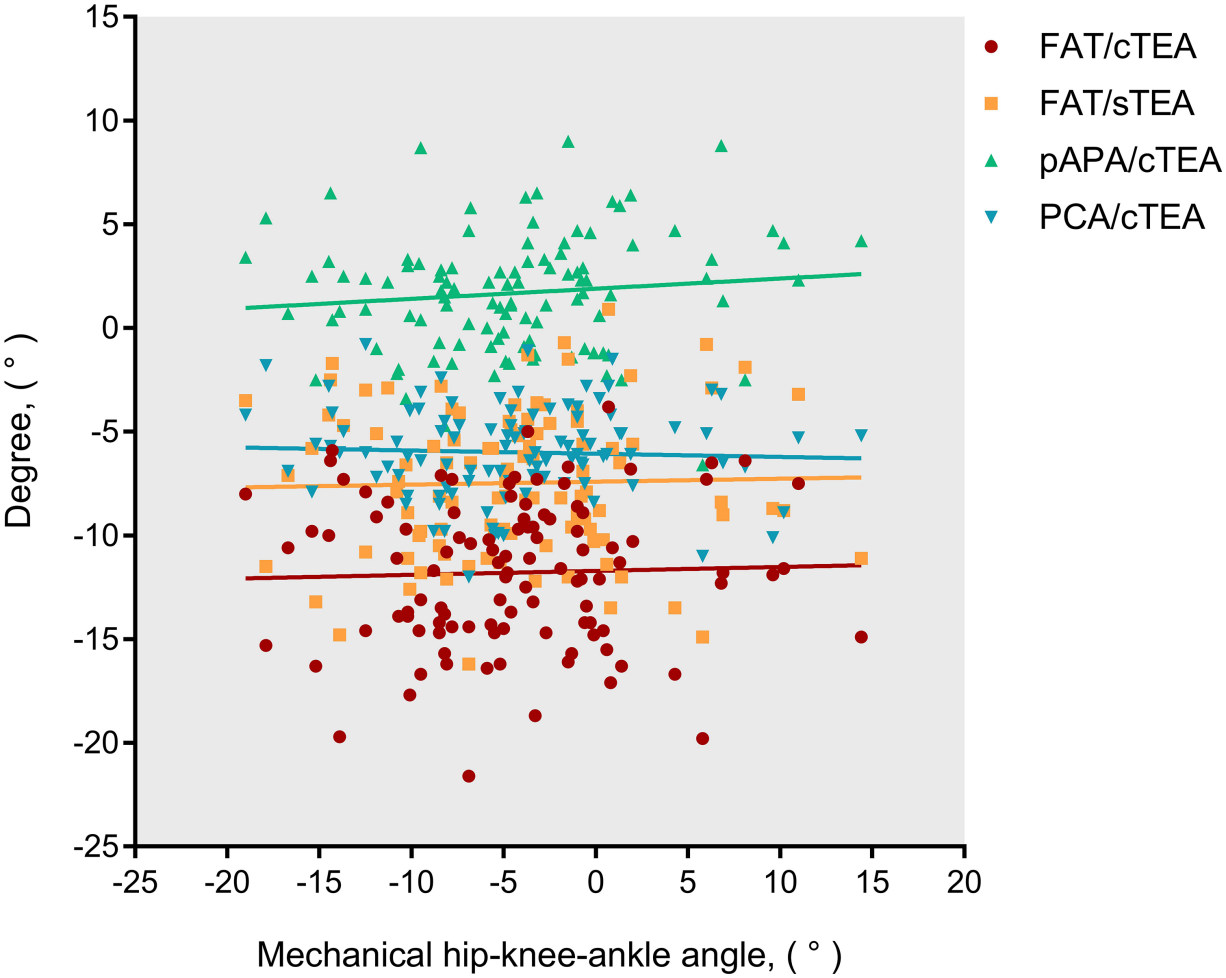
Scatter distribution and correlation between the distal femoral rotational angles and the mHKA.

The FAT/cTEA angle and the FAT/sTEA angle shared a frequency distribution pattern similar to that of the pAPA/cTEA angle and the PCA/cTEA angle (Figure 2). The interquartile ranges (*P*_75_–*P*_25_) of the FAT/cTEA angle and the FAT/sTEA angle were 5.5° and 5.4°, respectively, demonstrating greater variance when compared with the pAPA/cTEA angle and the PCA/cTEA angle, of which the interquartile ranges were 4.0° and 3.0°, respectively. By Pearson correlation analysis, no significant correlation was found between the femoral rotational reference angles and the mHKA (Figure 3). In terms of the mHKA, the correlation coefficient *r* of the FAT/cTEA angle, the FAT/sTEA angle, the pAPA/cTEA angle, and the PCA/cTEA angle were 0.034, 0.026, 0.110, and −0.045, respectively.

To evaluate the influence of knee osteoarthritis on the femoral rotational reference angles, we divided the study population into different subgroups according to the K–L grades and the JLCA absolute value (Table 3). A higher K–L grade and greater JLCA absolute value indicated severer degree of osteoarthritis (21). There was no statistical difference between the subgroups based on the K–L grades. The mean value of PCA/cTEA angle was −4.9° ± 1.8° when the JLCA absolute value was greater than 6°, significantly smaller than the |*x*| < 2° JLCA group (−6.3° ± 2.3°) and 2° ≤ |*x*| < 4° JLCA group (−6.4° ± 2.0°) (*P* < 0.05). Other rotational reference axes did not show statistical significance when compared within different JLCA subgroups.

**Table 3:** Subgroups’ analysis according to degree of knee osteoarthritis and constitutional lower limb alignment^a^.

Subgroups, *n* (%)		Distal femoral rotation angles			
		FAT/cTEA	FAT/sTEA	pAPA/ cTEA	PCA/cTEA
Subgroups according to the K–L grades					
0–1	17 (16.0%)	−12.8 ± 3.9	−8.2 ± 3.5	1.9 ± 2.6	−6.7 ± 2.3
2	14 (13.2%)	−11.2 ± 3.3	−6.6 ± 3.6	2.0 ± 3.3	−6.6 ± 2.3
3	31 (29.2%)	−11.9 ± 3.6	−7.5 ± 3.6	1.4 ± 3.2	−5.8 ± 2.1
4	44 (41.5%)	−11.6 ± 3.5	−7.4 ± 3.6	1.7 ± 2.6	−5.7 ± 2.2
Subgroups according to the JLCA^b^					
|*x*| < 2°	45 (42.5%)	−12.6 ± 3.5	−8.2 ± 3.3	2.0 ± 3.5	−6.3 ± 2.3*d
2° ≤ |*x*| < 4°	24 (22.6%)	−10.9 ± 3.5	−6.6 ± 3.9	1.0 ± 2.3	−6.4 ± 2.0*e
4° ≤ |*x*| < 6°	16 (15.1%)	−10.9 ± 3.1	−6.8 ± 2.8	1.4 ± 2.5	−6.0 ± 2.6
|*x*| ≥ 6°	21 (19.8%)	−11.7 ± 4.0	−7.5 ± 4.1	1.9 ± 2.2	−4.9 ± 1.8*de
Subgroups according to the aHKA^c^					
*x* ≤ −3°	42 (39.6%)	−12.4 ± 3.6	−8.1 ± 3.5	1.3 ± 2.7*f	−6.5 ± 2.5*g
−3° < *x* < 3°	49 (46.2%)	−11.4 ± 3.4	−6.9 ± 3.4	1.6 ± 2.6	−5.5 ± 1.8*g
*x* ≥ 3°	15 (14.2%)	−11.6 ± 3.8	−7.7 ± 4.0	3.1 ± 3.7*f	−6.1 ± 2.4

^a^ Data are presented as mean ± standard deviation (in degrees), negative value signifies internal rotation relative to the corresponding transepicondylar axis and positive value indicates external rotation.

^b^ The *x* value equals the JLCA value.

^c^ The *x* value equals the aHKA value.

* Statistical significance was found among subgroups that shared the same superscript letter (from d to g) (*P*-value < 0.05).

When patients were assessed according to the aHKA, the FAT line demonstrated greater stability than the pAPA and the PCA (Table 3). The pAPA/cTEA angle in the *x* ≥ 3° aHKA group was significantly greater than in the *x* ≤ −3° group (3.1° ± 3.7° vs. 1.3° ± 2.7°, *P *< 0.05), and the PCA/cTEA angle in the −3° < *x* < 3° aHKA group was smaller than in the *x* ≤ −3° group (−5.5° ± 1.8° vs. −6.5° ± 2.5°, *P* < 0.05). No statistical significance was found between different aHKA subgroups in terms of the FAT/cTEA angle and the FAT/sTEA angle.

## 4 Discussion

By comparing the angles formed by different indirect femoral rotational reference axes with the transepicondylar axes, our research suggested that the FAT line could be used as a reliable alternative reference axis for distal femoral rotational alignment. The FAT line was −11.8° internally rotated to the cTEA and −7.5° internally rotated to the sTEA; these features enable the FAT line to be used as an appropriate rotational reference axis during TKA. The newly designed prosthetic osteotomy tools can also refer to the FAT line (22, 23). The distribution pattern of the FAT/cTEA angle and the FAT/sTEA angle were similar to that of the pAPA/cTEA angle and the PCA/cTEA angle. Although the FAT/cTEA angle and the FAT/sTEA angle demonstrated a little greater variance than the pAPA/cTEA angle and the PCA/cTEA angle, the FAT formed angles were less affected by the degree of knee osteoarthritis and the variation of lower limb alignment, which were particularly diverse in patients with knee osteoarthritis.

The distal femoral anterior surface, which is adjacent to the upper edge of the femoral trochlear, is an easily exposed anatomical area and is often used as a reference landmark for femoral prosthesis size measurement in knee arthroplasty (24). The shape of the femoral anterior cortex surface is mostly flat or slightly concave in the middle, this configuration dramatically facilitates the FAT line measurement (17, 24). Compared with other rotational reference axes, the FAT line can be exposed without dissecting too much of the soft tissue and is still available in revision knee arthroplasty; hence such anatomical advantages deserve further exploration (22, 23).

Talbot and Bartlett first proposed that femoral component rotation alignment could refer to the FAT line (16). Since then, several studies investigating the FAT line have been published (17, 22, 23, 25). Watanabe et al. compared the FAT line and other rotational indicators on CT images in 150 patients with knee osteoarthritis. They found that the FAT/cTEA angle was −12.2° ± 3.6° and the pAPA/cTEA angle was −0.3° ± 3.6° (17). They subsequently invented a small jig to measure the FAT line during surgery. In their following study, the FAT/sTEA angle was −7.3° ± 4.0° on preoperative CT images, and the FAT line was −9.8° ± 3.2° internally rotated to the sTEA when measured during TKA surgery (23). The FAT line-related angles measured in the Watanabe et al. research studies were close to the values of our study. This might be because there was little articular cartilage at the anterior surface of the distal femur; hence, the CT-based and MRI-based measurements were indistinguishable. When it came to the pAPA/cTEA angle, of which the mean value in Watanabe’s research was smaller but the variance was greater than in our results, the subtle wear of trochlear cartilage might be detected by MR measurement, and the CT images could miss the lesion (26). Sathappan et al. also tried to assess the FAT line on MRI (27). In their study, the angle between the FAT line and the cTEA was −13.4° ± 3.4°. The variance of the angle was similar to ours, but the average value was greater than our results. It was unsure whether age could have an impact on the FAT measurement, because in their study, only normal knees were included and patients over 50 years of age were excluded.

Various degrees of cartilage wear, osteophyte hyperplasia, and bone-end deformity may occur in different stages of knee osteoarthritis, and these changes may lead to the alteration of the position of femoral rotational reference axes (28, 29). The posterior femoral condyles are in the load-bearing area of the femur, where cartilage wear is very common in patients with knee osteoarthritis, making the PCA susceptible to the deterioration of the knee joint circumstance (30). By analyzing more than 2,000 cases of femur CT data, Jang et al. pointed out that, when compared with other rotational reference axes, the external rotation of the PCA by 3° was the most accurate reference to the sTEA, but it had the highest intersubject variability (6). In accordance with previous studies, our study also found that the PCA manifested greater deviation in patients with severe knee osteoarthritis. Though no statistical difference was found, the PCA/sTEA angle in the K–L grade 4 subgroup was smaller than those in the K–L grade 0–1 and 2 subgroups. Such a phenomenon also existed in subgroups divided by the JLCA. The PCA/sTEA angle in the |*x*| ≥ 6° JLCA group was statistically significantly smaller than those with JLCA absolute value < 4°. By contrast, the FAT line and the pAPA remained relatively stable among the different K–L subgroups and JLCA subgroups.

Varus and valgus deformities of the lower extremities are common in patients with knee osteoarthritis. Such an abnormality of the lower limb alignment may exert influence on the indirect reference axes of distal femoral rotation. In the CT measurements by Watanabe et al. (17), a significant correlation was found between the PCA/cTEA angle and the femorotibial angle (correlation coefficient *r *= 0.31), while the FAT/cTEA angle and the pAPA/cTEA angle show no correlation to the femorotibial angle (*r *= 0.17, 0.07, respectively). Different from their results, our study, which was based on MRI, found no significant correlation between the femoral rotational reference angles and the mHKA. Such a disparity might be attributed to the distinctness of the research equipment. The MRI could easily identify the cartilage surface of the posterior femoral condyles and the epicondylar prominences, while the CT scans could not (18, 26).

The lower limb varus or valgus deformities may be caused by gradual development of the knee osteoarthritis, or be the result of constitutional alignment prior to the onset of osteoarthritis, or a consequence of the combination of the two factors (31). To assess the influence of the constitutional lower limb alignment on the indirect reference axes of femoral rotation, we introduced the concept of aHKA proposed by MacDessi et al. (19). The aHKA was mainly used to predict the constitutional alignment before the arthritis had developed. Surprisingly, patients with a valgus constitutional alignment (aHKA ≥ 3°) demonstrated a greater pAPA/cTEA angle than those with a varus constitutional alignment (aHKA ≤ −3°), and patients in a neutral constitutional alignment (−3° < aHKA < 3°) displayed a smaller PCA/cTEA angle than those with a varus constitutional alignment (aHKA ≤ −3°). These differences were statistically significant. On the contrary, the FAT/cTEA angle and the FAT/sTEA angle, both of which remained relatively stable among the different aHKA subgroups, seemed impervious to the constitutional alignment. Based on these findings, caution should be paid to the reliability of the rotational reference axes when attempting to restore the constitutional alignment during knee arthroplasty (32).

In addition, some researchers proposed that the trochlear anterior line (TAL) could also be used as an indirect reference axis for distal femoral rotation. The TAL, a little similar to the FAT line, is a line connecting the most anterior points of the medial and lateral femur condyles. Ji et al. used CT to compare the TAL and the FAT line in 76 patients who underwent TKA (33). In their study, the TAL/sTEA angle was −6.1° ± 2.5° and the FAT/sTEA angle was −9.5° ± 3.8°, the variance of the TAL was smaller than that of the FAT line. However, Ji’s study only included women patients who underwent TKA, and there was no data on the men. In another similar comparative study carried out by Nam et al., the TAL/cTEA angle was −5.1° ± 3.1° and the FAT/cTEA angle was −6.8° ± 6.1°, the mean value of the FAT/cTEA angle was significantly smaller than in other comparable studies, and the standard deviation was considerably larger (34). These differences might be attributed to their special measurement methods, which involved three-dimensional (3D) MR reconstruction and remodeling. Although the aforementioned two studies suggested that the TAL had better stability than the FAT line, other researchers had pointed out that the TAL/cTEA angle was positively correlated with the tibiofemoral angle and was affected by varus and valgus deformities of the lower extremities (35). Moreover, unlike the FAT line, which could continue to provide a reference during revision TKA, the TAL could only be used in primary TKA. The application comparison between the TAL and the FAT line warrants further research.

This study also had several limitations. First, we mainly used the cTEA as the benchmark to measure the angles formed with other indirect rotational reference axes. The sTEA served as the secondary reference axis, and only the FAT/sTEA angle was assessed. This choice was based on the fact that the cTEA could be detected in most of the knee joints and was relatively constant, while the sTEA was not apparent in all of the patients and had a probability of detection loss, which could hamper its clinical application. Second, our study only measured the rotational angles on MRI, and no CT data were included. MRI can identify the subtle changes of articular cartilage and reveal the soft tissue structure in a radiation-free mode. It is more prevalent in clinical practices to evaluate the knee joint circumstances. Most of our results were similar to the previously published CT-based studies, but there was some disparity in terms of certain indicators; Further study directly comparing the differences between the MRI and the CT scans is recommended. Finally, our study did not include some newly invented distal femoral rotational reference axes such as the TAL and the posterior cortical axis (34, 36, 37). Because these axes are still in the primitive stage of research and have not been widely used in practice, further comparative analysis would be appropriate when there is enough clinical application experience.

## 5 Conclusion

Appropriate alignment of the femoral prosthesis enables satisfying postoperative knee function. Placing the femoral component in a proper rotational alignment is one of the most challenging parts of TKA procedure. Though several rotational reference axes have been proposed to use preoperatively and intraoperatively, the results are imperfect because the axes can be covered by soft tissue or affected by cartilage wear. A combination usage of the PCA, the pAPA, and the transepicondylar axes is currently recommended to maximize the accuracy and diminish the variability. By comparing with the pAPA and the PCA on MRI, our study found that the FAT line was less affected by the degree of knee osteoarthritis and the variation of lower limb alignment. The FAT line could serve as a reliable candidate reference axis for distal femoral rotational alignment in total knee arthroplasty.

## Data Availability

The raw data supporting the conclusions of this article will be made available by the authors, without undue reservation.
